# A Study on the Classification Effect of sEMG Signals in Different Vibration Environments Based on the LDA Algorithm

**DOI:** 10.3390/s21186234

**Published:** 2021-09-17

**Authors:** Yanchao Wang, Ye Tian, Jinying Zhu, Haotian She, Hiroshi Yokoi, Yinlai Jiang, Qiang Huang

**Affiliations:** 1 School of Mechatronical Engineering, Beijing Institute of Technology, Beijing 100081, China; 3120170102@bit.edu.cn (Y.W.); 3120150094@bit.edu.cn (H.S.); qhuang@bit.edu.cn (Q.H.); 2 Beijing Advanced Innovation Center for Intelligent Robot and System, Beijing 100081, China; jinyingzhu@bit.edu.cn (J.Z.); yokoi@mce.uec.ac.jp (H.Y.); jiang.yinlai@uec.ac.jp (Y.J.); 3 Graduate School of Informatics and Engineering, The University of Electro-Communications, Tokyo 182-8585, Japan

**Keywords:** surface EMG signal, feature extraction, LDA algorithm, hand-motion recognition, vibration frequency

## Abstract

Myoelectric prosthesis has become an important aid to disabled people. Although it can help people to recover to a nearly normal life, whether they can adapt to severe working conditions is a subject that is yet to be studied. Generally speaking, the working environment is dominated by vibration. This paper takes the gripping action as its research object, and focuses on the identification of grasping intentions under different vibration frequencies in different working conditions. In this way, the possibility of the disabled people who wear myoelectric prosthesis to work in various vibration environment is studied. In this paper, an experimental test platform capable of simulating 0–50 Hz vibration was established, and the Surface Electromyography (sEMG) signals of the human arm in the open and grasping states were obtained through the MP160 physiological record analysis system. Considering the reliability of human intention recognition and the rapidity of algorithm processing, six different time-domain features and the Linear Discriminant Analysis (LDA) classifier were selected as the sEMG signal feature extraction and recognition algorithms in this paper. When two kinds of features, Zero Crossing (ZC) and Root Mean Square (RMS), were used as input, the accuracy of LDA algorithm can reach 96.9%. When three features, RMS, Minimum Value (MIN), and Variance (VAR), were used as inputs, the accuracy of the LDA algorithm can reach 98.0%. When the six features were used as inputs, the accuracy of the LDA algorithm reached 98.4%. In the analysis of different vibration frequencies, it was found that when the vibration frequency reached 20 Hz, the average accuracy of the LDA algorithm in recognizing actions was low, while at 0 Hz, 40 Hz and 50 Hz, the average accuracy was relatively high. This is of great significance in guiding disabled people to work in a vibration environment in the future.

## 1. Introduction

The hand is an important functional and motor organ of the human body. The inability to work due to upper limb motor dysfunction and limb deformity caused by various accidents imposes a great burden on the lives of patients, making them unable to work and earn their own living. The common method is to install artificial limbs to help them recover their hand function. In the current application of artificial limbs, myoelectric prosthesis [1] is the most widely used, which is characterized by strong sense of control and convenient use. Although artificial limbs can help people recover to a nearly normal life, whether they can adapt to severe working conditions is a subject that is yet to be studied. Generally speaking, the working environment is dominated by vibration. Therefore, this paper takes the gripping action as the research object, and focuses on the identification of grasping intentions under different vibration frequencies in different working conditions, so as to explore the possibility that patients with hand functional defects are engaged in different work vibration environments.

Surface Electromyography (sEMG) signals are a type of electrophysiological signal that has been widely studied and applied in clinical medicine and engineering [2]. It is a non-invasive procedure involving the detection, recording and interpretation of the electric activity of groups of muscles at rest (i.e., static) and during activity (i.e., dynamic). One important application of sEMG signals is their use in the control of prosthetic hands. However, in the prosthesis market, almost all sEMG controlled prosthetics are controlled outside a vibration environment, and there are few reports on the effects of different vibration environments on the sEMG signals of the upper limbs. Most of the studies focus on the effects of body vibration on the sEMG signals in the lateral thigh muscles [3,4,5,6,7]. The optimal vibration frequency (OVF), which corresponds to the maximal sEMG muscle response during whole-body vibration, between young, middle-aged, and older women in four muscles of the lower limbs was compared by Flaminia et al. [3]. The frequency corresponding to the maximal root mean square of the surface electromyogram (RMSmax) was found to be higher in older rather than younger women in all muscles. Giombini pointed out that the frequency of whole-body vibration (WBV) that elicits the greatest improvement in lower-limb power output after an acute exposure in older women should be prescribed in an individualized fashion, within the range of 30–35 Hz [8]. Marco Cardinale reported that the frequency, 30 Hz, as the one eliciting the highest reflex response in vastus lateralis muscle during WBVs in the half-squat position, would result in the highest sEMGrms [9]. Thus, in this paper, we focus on the influence of different vibration frequencies on the sEMG signals of the upper limbs.

sEMG signals, up to 6 mV and within a frequency range of 0–500 Hz, are one-dimensional time-series signals recorded by electrodes which are on the body skin over muscle bandage during neuromuscular system activity [10,11]. They present nonstationarity, nonlinearity, complexity, and large variation, which lead to difficulty in analyzing sEMG signals [12]. Therefore, it is difficult to identify the motion intention of upper limbs by sEMG signals. At present, the recognition of human movement intention through sEMG signals is mainly divided into three steps [13]: sEMG signal preprocessing, feature extraction and feature classification [14,15,16]. The sEMG signal preprocessing generally includes sEMG signal amplification, filtering and notch. In this process, noise removal is a significant step [17] that directly affects the authenticity of the signal. The next important step is feature extraction, which is performed to highlight the relevant structures in the sEMG signal and reject noise and unimportant sEMG signals. sEMG signal feature extraction mainly involves time-domain analysis, which includes the integrated EMG, mean absolute value (MAV), root mean square (RMS), and zero crossing (ZC) [18], frequency-domain analysis, which includes the autoregressive coefficient (AR) and modified median frequency (MMDF) [19], and time-frequency-domain analysis, which includes the wavelet transform (WT) and Wigner-Ville distribution (WVD) [20]. The more mature sEMG signal feature extraction method, which is safer and more reliable, is the time-domain analysis method because of its computational simplicity and wide use in research and in clinical practice. Hudgins et al. recognized four forearm motions by using five time-domain features, i.e., MAV, mean absolute value slope (MAVS), ZC, slope sign change (SSC) and waveform length (WL), and achieved an average accuracy rate of 91% [21]. Kim et al. successfully classified four wrist movements by using the integrated absolute value (IAV) and RMS [22]. The combination of different features is used for the characteristic classification of sEMG signals. Some feature classification algorithms such as linear discriminant analysis (LDA) [23], fuzzy logic, artificial neural networks (ANNs) [24], and the support vector machine (SVM) [25] have been used in several studies to assess the performance in classifying motion classes. Among them, the LDA classifier is much simpler to implement and much faster to train. Using LDA to reduce the dimensionality of the extracted feature signals, Chu et al. [26] identified nine movements of the forearm, wrist and palm with four electrodes and obtained an accuracy of 97.4%. When recognizing 10 independent and associated motions of fingers, Khushaba et al. used LDA to reduce a series of features to nine dimensions and achieved an accuracy of 90% [27].

Since sEMG signals are collected and extracted in the more complex external vibration environment, a simpler, safer and more reliable algorithm should be selected in the process of sEMG signal extraction and recognition to ensure the accuracy of signal recognition. In this research, the influence of various vibration frequencies on sEMG signals is studied by using the time-domain analysis method and LDA. The main aim of this study was to recognize the grasping and opening gestures in different vibration environments. This can be a good guide to evaluate whether it is suitable for disable people to work in such conditions, and help them return to work. First, we propose this new method using sEMG signal to recognize grasping and opening gestures toward to the real industry environment. Second, we develop the special system for the collection of sEMG signal for gesture recognition. Thirdly, we also make a full study on the recognition performance of different feature combination and vibration environment, so as to improve the performance of the system. This paper is organized as follows: Data acquisition from sEMG signals under a vibration environment and an illustration of how sEMG signals are converted to time series are introduced in Section 2. The main data processing algorithms, including feature extraction and feature classification, are introduced in Section 3. The results and a discussion are given in Section 4, and finally, the conclusions are presented in Section 5.

## 2. Data Acquisition

### 2.1. Data Platform Construction

**Figure 1:**
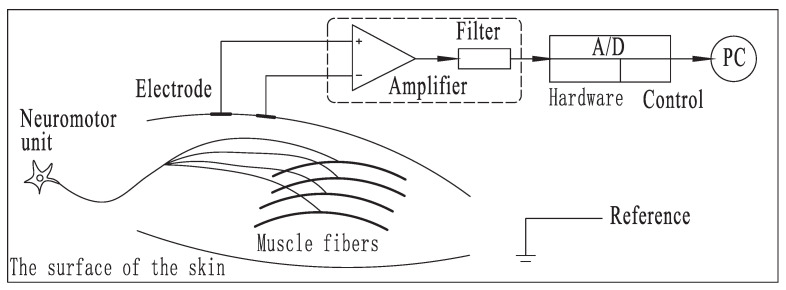
Sampling sEMG signals.

Based on the complex characteristics of sEMG signals, to ensure the authenticity of sEMG signals, a professional electrode, 42 mm × 26 mm, Ag/AgCl, type LT-7, which is produced by Shanghai LITU Medical Appliances Co., Ltd, was used in this paper. After the skin was wiped clean with medical alcohol or saline, the electrodes were tightly attached to the skin. The amplitude of the original sEMG signal collected by the electrode was only 0–6 mV. This signal was amplified by an amplifier circuit and then preprocessed to remove noise. Finally, a usable sEMG signal was obtained. Figure 1 shows the process of acquiring sEMG signals.

**Figure 2:**
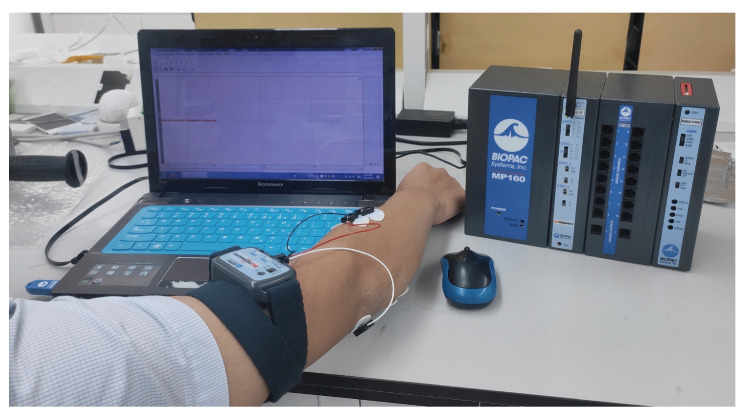
sEMG signal acquisition platform.

It is difficult to acquire sEMG signals stably. However, with the development of electronic technology, new detection and recording technologies have reached relative maturity, and many sEMG acquisition systems have been widely used in medical and scientific research fields, such as Trigno (produced by Delsys of the United States) and Myoscan (produced by Thought Technology of Canada). The equipment used to collect sEMG signals in this paper was the MP160 physiological record analysis system made by BIOPAC. The sEMG signal sampling frequency was 1000 Hz. Signal data transmission occurred via wireless Bluetooth. The sEMG signal detection platform established in this paper is shown in Figure 2. Based on our survey, the vibration frequency is often the same for a certain working environment in real life. In addition disabled people can often only do the same job, which usually requires a single vibration environment. Our training set and testing set are from the same frequency environment.

### 2.2. Acquisition of Signals at Various Vibration Frequencies

**Figure 3:**
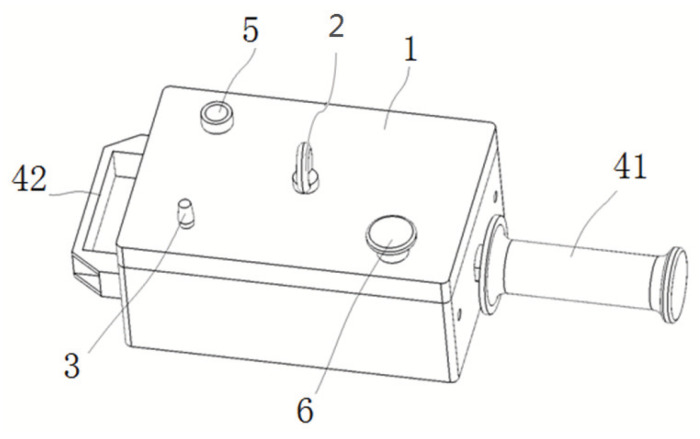
Vibration simulator (1, external shell; 2, hook; 3, frequency adjustment knob; 41, industrial handle; 42, commonly used handle; 5, switch button; 6, emergency stop button).

In everyday life, there are various kinds of vibration equipment, such as electric toothbrushes, household cleaners, hair dryers, and juicers. Of course, among the commonly used construction tools, there is also some equipment with larger vibration amplitudes, such as electric drills, polishing machines, and cutting machines which have mixed vibration. The range of vibration frequency is selected based on the engineering equipment we operate in the real industrial environment. Thus, how to simulate this kind of mixed vibration in the laboratory environment is the primary problem. Figure 3 presents the vibration simulator designed in this paper, which can simulate various vibration environments that contains the vertical and horizontal vibration within the frequency range of 0–50 Hz. In the design of the vibration simulator, the safety and reliability factors in the operation process are considered, and the hoisting hook, emergency stop button and other facilities are set up. At the same time, combined with most operating modes in daily construction, the operating handle and cooperating handle are set on both sides of the vibration simulator. Throughout the test, the vibration simulator was placed on a 750 mm high platform, which is a suitable height to subjects. Thus, it can be easy for subjects to grasp while standing. The vibration direction is mixed. At the same time, in order to approximate a real application, our experiment is based on a real operating environment in which subjects are required to operate vibration equipment with a comfortable grasp force.

**Figure 4:**
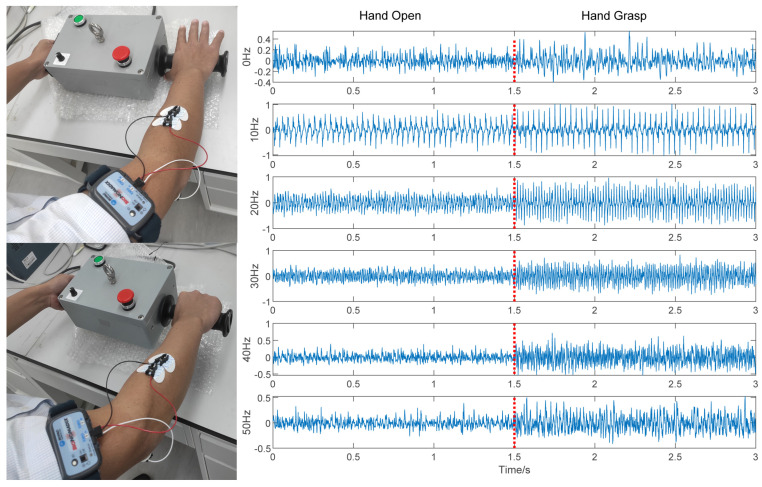
The sEMG signals for the open and grip modes of the prosthetic hand under various vibration frequencies.

In this system, after being amplified and filtered, the acquired sEMG signals are converted to digital signals by an analog/digital converter and transmitted to a PC to undergo signal processing for feature extraction. In this paper, the 0 Hz, 10 Hz, 20 Hz, 30 Hz, 40 Hz and 50 Hz vibration frequencies are selected as the frequencies to measure from the vibration frequency range of 0–50 Hz. The digital signals collected in each frequency environment, where the signals of each pattern last for 1.5 s, are shown in Figure 4. The sEMG signals generated during the different state transitions are removed. In total, we collected 132 samples of each individual for experiment. The ratio of the training set to testing set is 1/1, and these two sets are collected at different times to avoid overfitting.

### 2.3. Signal Window Processing

**Figure 5:**
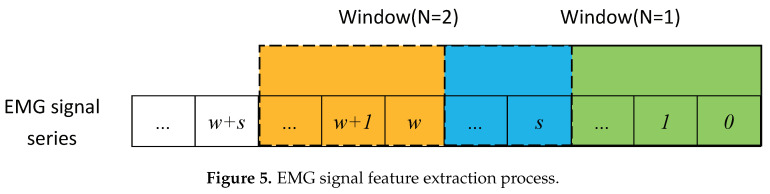
EMG signal feature extraction process.

The selection of signal sampling points is always limited, and the computer processes the signal data using the windowed overlapping algorithm. The algorithm iteratively extracts the eigenvectors of a sequence of signal data by intercepting the nearest signal fragment. This process is shown in Figure 5, where a window is used to generate the raw samples. To be specific, for each instance of recognition, the segment of signals in the current window is cut from the sEMG series to compose a raw sample for recognition, where the width of window *w* determines the length for each sample. Then, the window moves forward *s* steps, and a new raw sample is obtained. The real-time recognition is realized by repeating this process successively. Thus, the period between two adjacent instances of recognition is obtained by $T_{r}=1/f_{r}=s/f_{s}$, where $f_{s}$ is the frequency of signal sampling. In Figure 5, when N=1, the signals in the current window are Data(0) − Data(w−1), and when N=2, Data(*s*) − Data(w+s−1), where Data(*x*) is the signal acquired at time $x/f_{s}$ and N=k corresponds to the *k*th instance of recognition. The original signal waveforms are shown in Figure 4. Since the sampling frequency of the signal in this paper is 1000 Hz, the window size should be as small as possible to ensure smooth and accurate signal extraction, and the signal sliding increment needs to be reasonably set. In this paper, the optimal window size and optimal movement increment are verified through supervised training to ensure that each signal can be extracted at various frequencies and the correct rate of action classification is the highest.

## 3. Algorithm Description

Since this paper mainly studies the effects of sEMG signal classification and recognition under environments of various vibration frequencies, the external environment is more complex. Considering the safety and reliability factors comprehensively, and by combining the existing signal processing means, in this paper, we extract signal feature vectors by various time-domain analysis algorithms, and the grasping and opening modes of the hand are classified by the LDA algorithm. The relevant algorithms are introduced from the following two aspects: signal feature extraction and motion classification.

### 3.1. Feature Extraction Methods

**Figure 6:**
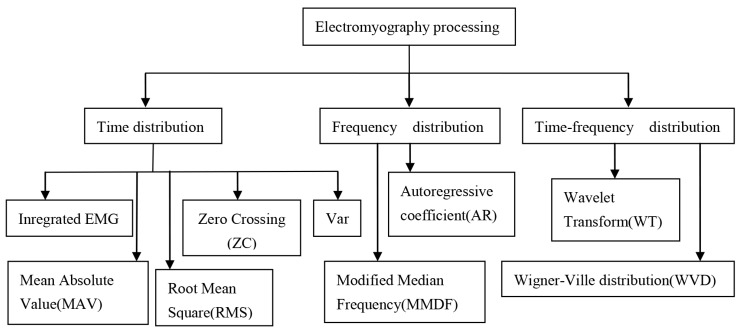
An overview of several sEMG signal processing algorithms.

SEMG signal processing can be divided into three categories: time domain, frequency domain, and time-frequency domain. These approaches make it easier for researchers to characterize sEMG signals. Figure 6 provides an overview of the sEMG signal processing methods that are introduced in this paper.

Among them, the integral myoelectric value, defined in Equation (1), the RMS value, defined in Equation (2), and the MAV, defined in Equation (3), can reflect the variation characteristics of the sEMG signal amplitude in the time domain.

(1) $$iEMG=\int_{t}^{t+T}\left|EMG(t)\right|dt\;$$

(2) $$RMS=\sqrt{\frac{1}{N}\sum_{n=1}^{N}{x_{n}}^{2}}$$

(3) $$MAV=\frac{1}{N}\sum_{n=1}^{N}\left|x_{n}\right|$$

where *N* = number of samples in a signal segment and $x_{n}$ is the *n*th sample in the sEMG signal.

*ZC* is the number of times the voltage amplitude of an sEMG signal crosses the 0-*y*-axis, i.e., the number of times the amplitude of the signal exceeds the zero-amplitude level due to low voltage fluctuation or background noise. To avoid the influence of low voltage fluctuation or background noise, the threshold condition is applied to the signal amplitude, and the following formula is obtained:

(4) $$ZC=\sum_{i=1}^{N-1}\left[sgn\left(x_{n}\times x_{n-1}\right)\cap \left|x_{n}-x_{n-1}\right|\geq threshold\right];sgn\left(x\right)=\left\{\begin{array}{cc} 1, & \mathrm{if}x\geq threshold\\ 0, & \mathrm{otherwise}. \end{array}\right.$$

Variance (*VAR*) is a measure of the degree of dispersion in a random variable or a set of data. Probability variance is used to measure the degree of deviation between a random variable and its mathematical expectations, known as the mean. Thus, for a set of random variables, for *N* samples taken at random, the variance of this set of samples is $X_{i}$ squared divided by *N* minus 1.

(5) $$VAR=\frac{\sum_{i=1}^{n}{\left(X_{i}-\bar{X}\right)}^{2}}{n-1}.$$

where $X_{i}$ represents the *i*th sample, $\bar{X}$ represents the population mean of the sample data and *n* represents the total number of samples.

In the aspect of frequency-domain analysis, the main analysis method is to apply the fast Fourier transform to the sEMG signals to obtain their spectrum or power spectrum, which can reflect the changes in the sEMG signals in different frequency components.

In frequency-domain analysis, the AR time-series model, where the coefficients are linearly combined and formulated as estimations of previous samples, is one of the most commonly used methods to analyze sEMG signals. An equation for the *AR* coefficient is shown in Equation (5).

(6) $$AR=\bar{x}\left(k\right)=\sum_{i=1}^{P}a_{j}\left(i\right){\bar{x}}_{j}\left(k-i\right)+\mu_{k},\;\;k=0,1,2...$$

where $\bar{x}\left(k\right)$ denotes the signal, $a_{j}$ represents the *AR* coefficients, *p* is the order of the AR model, and $\mu_{k}$ is the white noise sequence.

To a certain extent, the mean frequency obtained by the short-time Fourier transform (STFT) can better represent the fatigue changes of muscles. The formula for calculating the median frequency is shown in Equation (6).

(7) $$\int_{f_{1}}^{MDF}PS(f)df=\int_{MDF}^{f_{2}}PS(f)df$$

where PS(f) is the spectrum of the sEMG signal and $f_{1}$, $f_{2}$ is the frequency range of the sEMG signal.

The STFT introduced by Gabor [28] retains the time information of the traditional Fourier transform through a window of fixed size. The WT is the solution of the high time-frequency characteristic obtained by introducing the variable window. The WT is defined mathematically as shown in Equation (7).

(8) $$\langle f,\Psi_{a,b}\rangle =\frac{1}{\sqrt{a}}\int f\left(t\right)\Psi \left(\frac{\bar{t-b}}{a}\right)dt\;$$

where Ψ(t) is the mother wavelet, which is scaled by factor a and delayed by time *b*, and f(t) is the signal for analysis.

The WVD was first introduced by Wigner in the area of quantum mechanics in 1932, and later in 1948, Ville developed and applied the same transformation to signal processing and spectral analysis [29]. Equation (8) shows the formula for the WVD.

(9) $$W_{x}\left(t,f\right)=\int_{-\infty }^{\infty }x\left(t+\frac{\tau }{2}\right)x^{*}\left(\tau -\frac{\tau }{2}\right)e^{-2j\pi f\tau }d\tau \;$$

where $x\left(t+\frac{\tau }{2}\right)x^{*}\left(\tau -\frac{\tau }{2}\right)$ is the instantaneous autocorrelation function and * denotes the conjugate operation.

In addition to the processing techniques listed in this paper, there are several techniques such as the Hilbert-Huang transform [30] and S-transform [31] that have been used to analyze sEMG signals. Currently, the sEMG signal processing algorithms used by sEMG prostheses are mainly the integrated EMG, RMS value, and median frequency due to their good qualities, including linearity, high efficiency, and simplicity.

### 3.2. Feature Classification

Many mature algorithms for feature classification are commonly used at present, such as LDA, principal component analysis (PCA), the SVM, K-nearest neighbors (KNN), the ANN, and decision trees. LDA is a classic, simple and practical method for dimensional reduction, first proposed by Fisher [32] in 1936 for the dichotomy problem, also known as Fisher’s linear discrimination. The basic idea of the LDA classifier is to find an optimal discriminant vector space to maximize the ratio of the interclass dispersion to intraclass dispersion of samples projected into a space.

The calculation steps of the LDA algorithm are as follows:

(a) The mean vector for each class is defined to be:

(10) $$u_{i}=\frac{1}{n_{i}}\sum_{x\in class_{i}}x$$

where $n_{i}$ is the number of samples of each class and *x* denotes the original feature vectors of each class.

(b) The population mean of the sample is calculated as:

(11) $$u=\frac{1}{m}\sum_{i=1}^{m}x_{i}$$

where *m* is the total number of classes.

(c) The interclass divergence matrix and the intraclass divergence matrix are calculated as:

(12) $$S_{b}=\sum_{i=1}^{c}n_{i}\left(u_{i}-u\right){\left(u_{i}-u\right)}^{T}$$

(13) $$S_{w}=\sum_{i=1}^{c}\sum_{x\in class_{i}}\left(u_{i}-x_{k}\right){\left(u_{i}-x_{k}\right)}^{T}$$

Here, it is important to note that a weighted average is needed when calculating the total $S_{b}$ and $S_{w}$, as the number of samples for each class may be different.

(d) Finally, the Fisher criterion can be expressed in terms of $S_{b}$ and $S_{w}$ as:

(14) $$J\left(w\right)=\frac{w^{T}S_{b}w}{w^{T}S_{w}w}$$

By maximizing the generalized Rayleigh quotient, we can use the Lagrange multiplier method [33] to find the required eigenvectors. Then, the original feature matrix (mxn) is projected by:

(15) $$y=w^{T}x$$

The matrix *y* represents the eigenvector of the projection of the original data, through which we can obtain the best recognition accuracy.

## 4. Data Analysis and Processing

### 4.1. Window Size Selection

**Figure 7:**
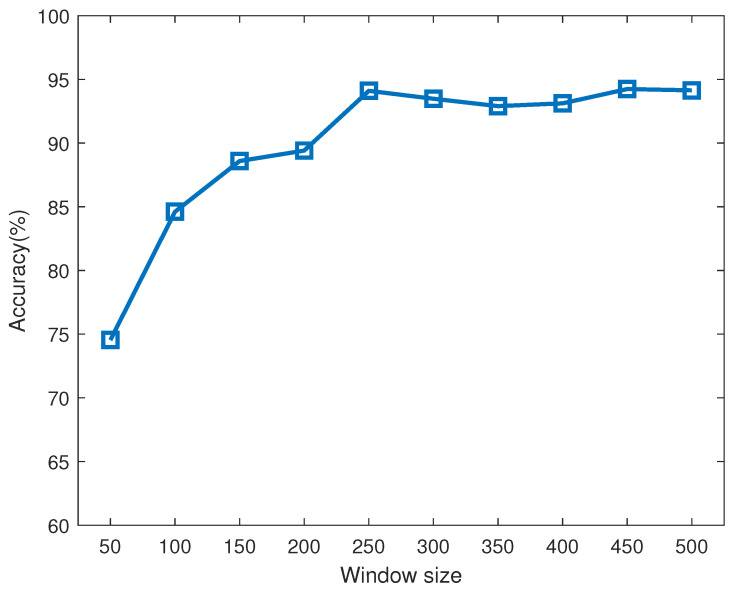
Optimized selection of sliding window size.

Through the established experimental platform, the 0–50 Hz vibration environment can be simulated, among which five vibration environments of 10 Hz, 20 Hz, 30 Hz, 40 Hz and 50 Hz are selected for testing.The sampling frequency of the sEMG signal is 1000 Hz. A suitable sliding window size can improve not only the algorithm speed but also the accuracy of algorithm recognition. If the window is greater than 300 ms, the characteristic value of the calculated sEMG signal will be too large in terms of the time scale, which will cause people to feel frustrated. Thus, the time from muscle contraction to the system producing the corresponding classification decision should not be less than 300 ms. The curve in Figure 7 shows the recognition accuracy of the algorithm under sliding windows of different sizes. Clearly, the best effect is achieved when the size of the sliding window is set to 250 ms.

On the other hand, setting the window too small will place a high computational burden on the system and affect the accuracy. Due to the nonlinear nature of sEMG signals, it is very difficult to make a correct decision through a window. Moreover, by making as many decisions as possible, you can smooth those decisions and obtain the final decision. The overlapping windowing scheme can solve these problems. The number of windows can be calculated as:

(16) $$No.\;of\;windows=\frac{data\;length-window\;size}{window\;increment}+1$$

In this paper, the window length is set to 250 ms, and the window increment is set to 100 ms. Out of 5000 data points, 48 eigenvalues are eventually obtained.

### 4.2. Combinatorial Optimization Design of Two-Feature Classification

In this paper, 5000 points of data are intercepted for each action, and the time-domain features are extracted. The LDA algorithm is used to classify two actions, i.e., opening and grasping, of the hand under different vibration frequencies. Two types of features of each action are randomly selected as the input parameters of the LDA algorithm. In this paper, six time-domain eigenvalues (MAV, VAR, RMS, ZC, MIN, MAX) are used as input. The total number of eigenvalue combinations is 15.

**Figure 8:**
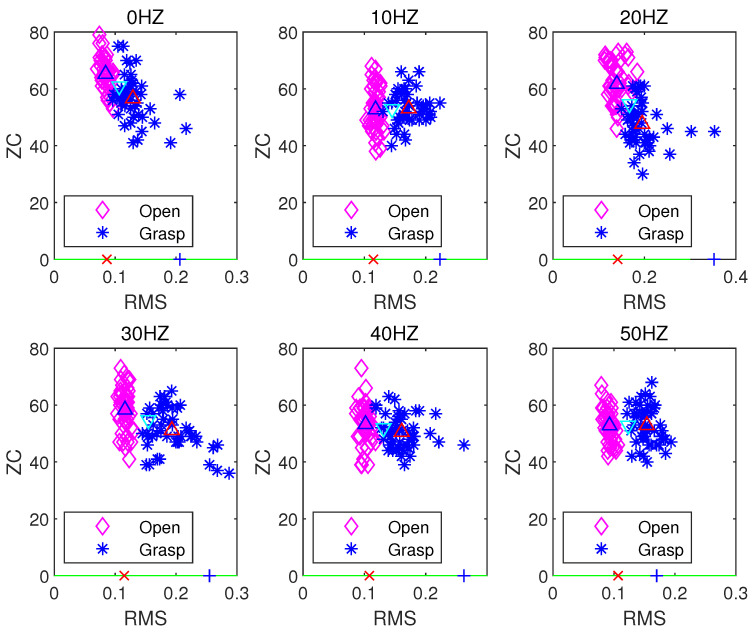
LDA classification based on the ZC and RMS feature extraction algorithm.

**Figure 9:**
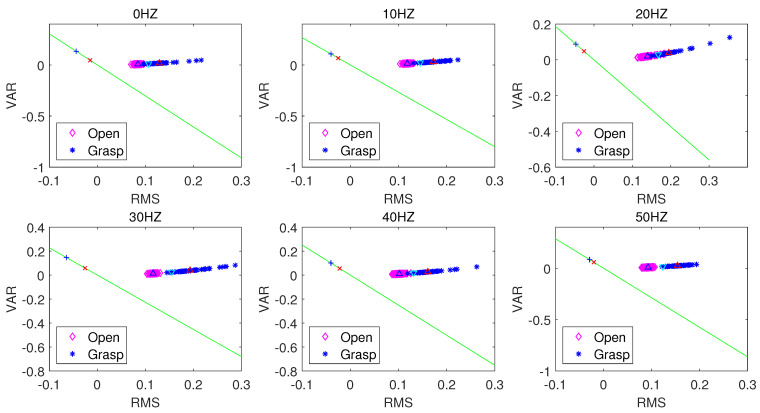
LDA classification based on the VAR and RMS feature extraction algorithm.

**Figure 10:**
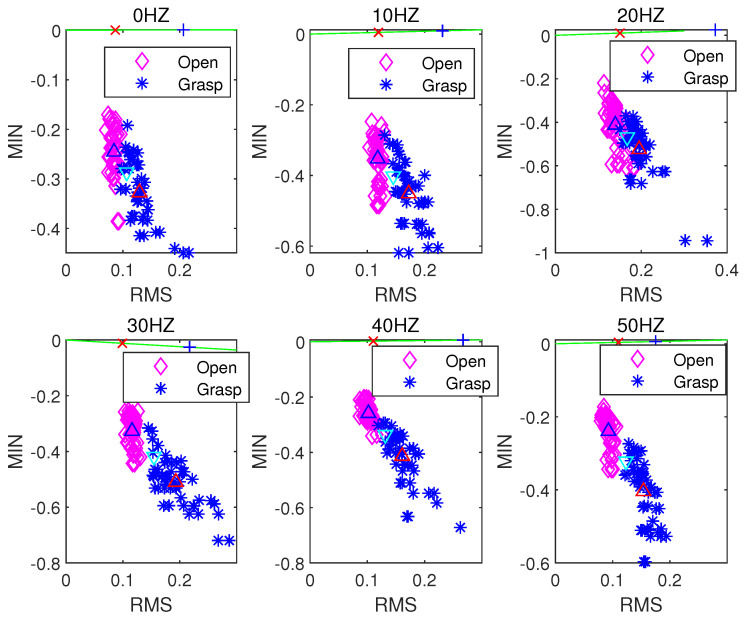
LDA classification based on the MIN and RMS feature extraction algorithm.

Figure 8, Figure 9 and Figure 10 respectively present the recognition of sEMG signals in the opening and grasping states of a hand by the LDA algorithm when using the ZC and RMS eigenvalues, VAR and RMS eigenvalues, and MIN and RMS eigenvalues in combination. In the above three figures, the blue upper triangle and the red upper triangle represent the mean values of the internal eigenvalues of the opening and grasping states of the hand, respectively. The lower cyan triangle represents the average of all eigenvalues. The green lines represent the best projection lines that can distinguish different features (classes). The red crosses and blue plus signs are the features after projection for open and grasp respectively. The LDA algorithm is a supervised learning algorithm. After learning the training data, we test the corresponding unknown classified action data, obtain a group of data lists, and calculate its accuracy. The test accuracy is formulated as:

(17) $$Accuracy=\frac{Total\;No.\;of\;features-No.\;of\;feature\;classification\;errors}{Total\;No.\;of\;features}\times 100\%$$

The LDA algorithm is used to train and learn the sEMG signal data of the two motion modes under different vibration frequencies. In this paper, six different metrics for extracting the time-domain eigenvalues are selected, i.e., MAV, VAR, RMS, ZC, MIN, an MAX, with combinations of these characteristic values (15 in total) selected as input to the LDA algorithm.

**Figure 11:**
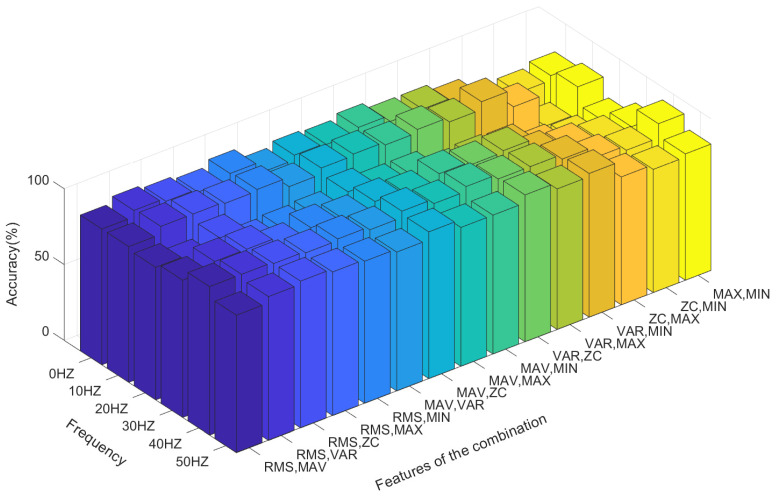
The recognition accuracy of each feature combination for the LDA algorithm.

Table 1 shows the accuracy of the action state recognition of the grasping and opening of human hands in a 0–50 Hz vibration environment when selecting any two time-domain features as input signals through the LDA algorithm under a sampling frequency of the sEMG signal of 1000 Hz, a signal processing window size of 250 ms, and a step size increment of 100 ms. Clearly, when the RMS and ZC eigenvalues are used as the classification characteristic signals, the LDA algorithm has the best accuracy in terms of pattern recognition of the opening and gripping movements of hands at different vibration frequencies. When the RMS and VAR, and RMS and MIN eigenvalues are used as classification inputs, the classification effect is also relatively good. Their accuracy is poor (i.e., less than 90%) only at a vibration frequency of 20 Hz. However, when ZC and MAX, ZC and MIN, and MAX and MIN are combined as inputs, the accuracy of the classification action recognition of the LDA algorithm is poor, with the lowest accuracy being only 63%. The distribution state of the accuracy can be observed more intuitively through Figure 11.

### 4.3. Combinatorial Optimization Design of Three-Feature Classification

**Figure 12:**
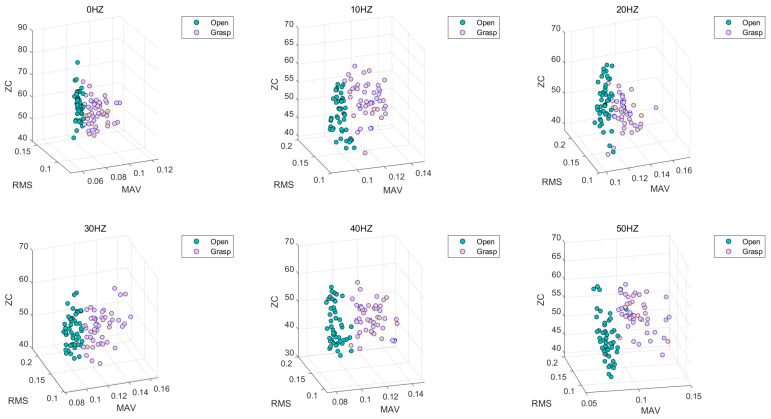
LDA classification based on the RMS, MAV and ZC feature extraction algorithm.

When three eigenvalues are combined for the LDA algorithm, a total of 20 combinations can be generated from the six eigenvalues. Figure 12 shows the classification statuses of MAV, RMS, and ZC when the three eigenvalues are used as input. The green data point represents the eigenvalue when the hand is open, and the pink data point represents the eigenvalue when the hand is in the grasp state. Figure 11 shows that when three features are used as the input of the LDA classification algorithm, the opening and grasping movements of the hand are easy to distinguish.

**Figure 13:**
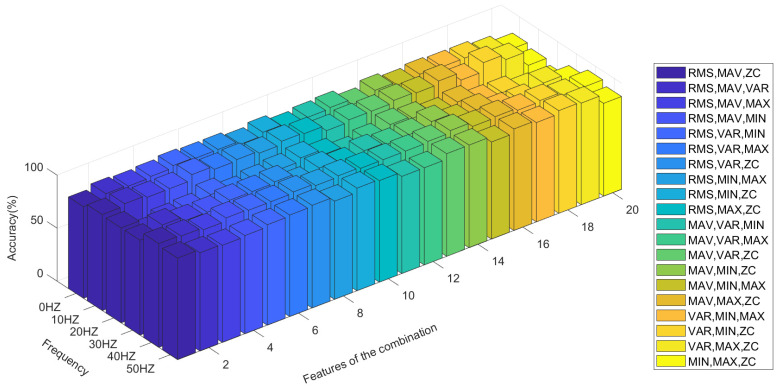
The recognition accuracy of all three-feature combinations for the LDA algorithm.

Table 2 shows the classification accuracy of the LDA algorithm for hand opening and hand grasping at different vibration frequencies from 0–50 Hz when three different time-domain eigenvalues are used as the input conditions. Figure 13 can more intuitively show the accuracy of the LDA algorithm at different vibration frequencies. By comparison, it can be seen that when RMS, MIN, VAR and RMS, MIN, ZC are respectively input into the LDA algorithm as feature combinations, the classification accuracy is relatively high. The accuracy is low only at the 20 Hz vibration frequency, with a minimum accuracy of 88%, while a maximum accuracy rate of 98% is achieved in the rest of the vibration environments, with the rate always exceeding 90%. By contrast, when MAX, MIN, VAR and MAX, MIN, ZC are input into the LDA algorithm as feature combinations, the classification accuracy is relatively low.

### 4.4. Combinatorial Optimization Design of Classification Using All Features as Input

When all the selected time-domain features in this paper are used as the input conditions for the classification of actions by the LDA algorithm, the final accuracy of the classification of the hand opening and grasping actions is as shown in Table 3. Clearly, we can see that our method can always keep a high accuracy from the frequency of 0 Hz to 50 Hz. Overall, the accuracy for the frequencies of 0 Hz, 10 Hz, 40 Hz, 50 Hz are often higher than that of 20 Hz and 30 Hz.

### 4.5. Comparison of Average Accuracy

In the 0–50 Hz vibration environment, to more intuitively represent the accuracy of the action classification for each characteristic value combination in the LDA algorithm, this paper uses the average accuracy, obtained by the following formula.

(18) $$Average\;accuracy=\frac{1}{m}\sum_{i=1}^{m}{\left(Accuracy\right)}_{i}$$

where *m* represents the total number of each two-feature combination of the time-domain features.

**Figure 14:**
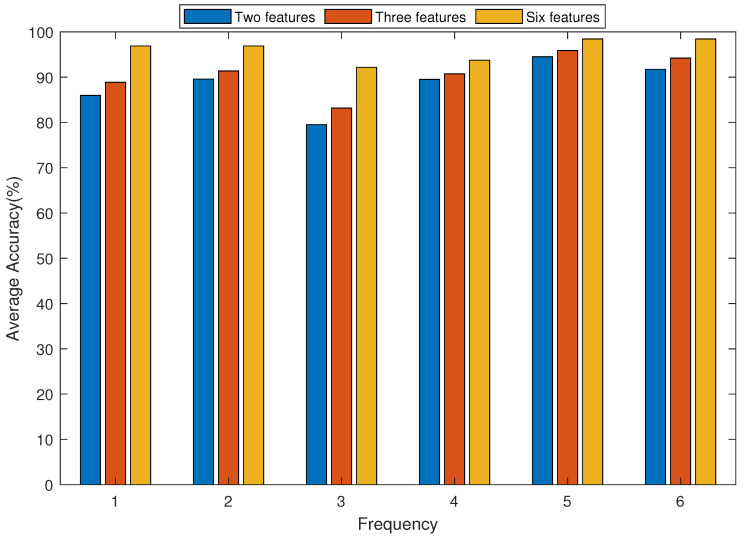
Accuracy comparison under different numbers of features for the LDA algorithm.

Figure 14 shows the average accuracy of LDA algorithm classification under six different vibration environments, namely, 0 Hz, 10 Hz, 20 Hz, 30 Hz, 40 Hz and 50 Hz, when two, three and all six eigenvalues are used as the input conditions of the LDA algorithm. It can be seen from the figure that with the increase in the number of input conditions of the LDA algorithm, the classification accuracy of the LDA algorithm gradually increases, that is, the more types of input eigenvalues there are, the higher the accuracy. At the same time, it can be seen that the classification accuracy achieved by the LDA algorithm is low at a vibration frequency of 20 Hz relative to that at 0 Hz, 40 Hz and 50 Hz.

Feature importance on recognition accuracy in different vibration conditions.

As discussed above, we illustrate the recognition accuracy when only using two and three features for classification. Through the results, we can find an interesting thing when observing the contribution in accuracy. There are two factors that will influence the recognition accuracy, namely the feature number and feature quality, respectively. When the feature number increases, the average recognition follows, which is a well-known method for accuracy improvement.

We define the feature importance or feature contribution for the second finding. Based on our comparison, we find that combinations with some special features may often have high and low recognition accuracy, whatever the vibration frequency is. For example, it is general considered that RMS should be a feature of good quality, which means that if a combination contains this feature, the accuracy will always be better. This can be seen in the recognition results with two feature combinations, as well as three feature combinations. As Table 1 and Table 2 show, the combinations containing this feature are always able to show a high accuracy, whatever the vibration frequency is. In contrast, MIN and MAX should be worse features, and the combination which contains them may show a lower accuracy. There is no denying that when the number of features increases, these positive or negative influences will be weakened.

So, the better way to enhance accuracy in the future is to select the features with high contribution and avoid features with worse performance, which can be a good method to guide us for good application without complex research.

## 5. Conclusions

In this paper, we aimed to find a suitable scheme to improve the recognition performance of grasping and opening in an industrial environment, and thus provided a potential guide for disabled people who want to work well in different vibration environments. We mainly studied an industry situation where disabled people work only with simple gestures. Also, to allow disabled people to work freely, it may only be necessary for them to hold and control a machine, e.g., with grasp and opening movements. Our main aim is testing whether people can work in a particular vibration situation. To improve the reliability of hand-motion intention detection and reduce the computational complexity, this paper adopts a structure combining the time-domain features and LDA algorithm to identify opening and grasping hand motions under a vibration environment. In this paper, through a comparative test, it is determined that the LDA algorithm is the most accurate for human-motion-intention recognition when the sEMG signal feature extraction window size is 250 ms and the window increment is 100 ms. By a comparison of the number of eigenvalues input into the LDA algorithm, it is found that the greater the number of eigenvalues is, the higher the accuracy of hand-motion recognition by the LDA algorithm. When the number of input eigenvalues reaches 6, the accuracy of action recognition is the highest, up to 98.4%. At the same time, under a vibration frequency of 20 Hz, the sEMG signal is greatly affected by the vibration environment, and the accuracy of action recognition by the LDA algorithm is relatively low, only 88.5%, while at 0 Hz, 40 Hz and 50 Hz, the accuracy of action recognition by the LDA algorithm is relatively high.

To further study the impact of the vibration environment on sEMG signals, the following future research directions will be taken:

(1) Enlarge the vibration frequency of the vibration environment and study the influence of a wider range of vibration frequencies on sEMG signals.
(2) Study the accuracy of the LDA algorithm in terms of human intention recognition by comparing the time-domain and frequency-domain characteristics of sEMG signals.
(3) Further compare the classification effects of various classifiers, such as the SVM, fuzzy logic, and the ANN.

## Figures and Tables

**Table 1:** Classification accuracy (%) under several combinations of two different time-domain features.

No.	Subject	0 Hz	10 Hz	20 Hz	30 Hz	40 Hz	50 Hz
1	ZC, RMS	91.67	94.79	90.63	94.79	96.88	96.88
2	VAR, RMS	93.75	94.79	82.29	94.79	97.92	94.79
3	MIN, RMS	93.75	94.79	80.21	92.71	96.88	93.75
4	MAV, RMS	89.58	89.58	87.50	90.63	97.96	90.63
5	MAX, RMS	89.58	93.75	79.17	88.54	95.83	94.79
6	MAV, VAR	86.46	88.54	81.25	90.63	94.79	91.67
7	MAV, ZC	89.58	92.71	88.54	93.75	98.96	96.88
8	MAV, MAX	88.54	93.75	83.33	90.63	95.83	91.67
9	MAV, MIN	91.67	91.67	83.33	95.83	98.96	91.67
10	VAR, ZC	87.5	93.75	77.08	93.75	95.83	96.88
11	VAR, MAX	87.5	90.63	78.13	94.79	95.83	92.71
12	VAR, MIN	83.33	95.83	77.08	84.38	92.71	94.79
13	ZC, MAX	65.63	84.36	68.75	81.25	85.42	84.38
14	ZC, MIN	73.96	63.54	66.67	78.13	82.29	81.25
15	MAX, MIN	77.08	81.25	69.79	78.13	91.67	83.33

**Table 2:** Classification accuracy (%) under combinations of three different time-domain features.

No.	Subject	0 Hz	10 Hz	20 Hz	30 Hz	40 Hz	50 Hz
1	RMS, MAV, ZC	86.46	90.63	89.58	89.58	97.92	95.83
2	RMS, MAV, VAR	90.63	92.71	76.04	95.83	96.88	92.71
3	RMS, MAV, MAN	89.58	90.63	81.25	91.67	94.79	91.67
4	RMS, MAV, MIN	90.63	90.63	82.29	91.67	97.79	92.71
5	RMS, VAR, MIN	92.71	95.83	88.54	94.79	96.88	94.79
6	RMS, VAR, MAX	91.67	92.71	82.29	94.79	96.88	95.83
7	RMS, VAR, ZC	91.67	93.75	85.42	95.83	97.92	96.88
8	RMS, MIN, MAX	88.54	91.67	87.5	86.46	95.83	92.71
9	RMS, MIN, ZC	91.67	94.79	90.63	92.71	96.88	96.88
10	RMS, MAX, ZC	87.5	93.75	85.42	88.54	95.83	95.83
11	MAV, VAR, MIN	89.58	88.54	79.17	92.71	95.83	91.67
12	MAV, VAR, MAX	90.63	90.63	83.33	90.63	95.83	91.67
13	MAV, VAR, ZC	85.42	90.63	89.58	91.67	96.88	96.88
14	MAV, MIN, ZC	90.63	92.71	87.5	95.83	98.96	96.88
15	MAV, MIN, MAX	87.5	91.67	83.33	88.54	94.79	91.67
16	MAV, MAX, ZC	89.58	94.79	86.46	90.63	95.83	95.83
17	VAR, MIN, MAX	88.54	87.5	79.17	83.33	93.75	93.75
18	VAR, MIN, ZC	88.54	95.83	79.17	83.33	94.79	96.88
19	VAR, MAX, ZC	85.42	90.63	73.96	91.67	91.67	95.83
20	MIN, MAX, ZC	80.21	77.08	72.92	84.38	91.67	87.5

**Table 3:** Classification accuracy (%) under all time-domain features.

No.	Subject	0 Hz	10 Hz	20 Hz	30 Hz	40 Hz	50 Hz
1	All Features	96.88	96.88	92.19	93.75	98.44	98.44

## Data Availability

The data presented in this study are available on request from the corresponding author.

## Abbreviations

The following abbreviations are used in this manuscript:

sEMG	surface electromyographic
LDA	linear discriminant analysis
RMS	root mean square
ANN	artificial neural network
MAV	mean absolute value
ZC	zero crossing
VAR	variance
